# Winner takes all? Understanding the impact of energy transition on employment under the constraint of China’s carbon peak target

**DOI:** 10.1186/s13021-026-00475-9

**Published:** 2026-06-23

**Authors:** Jianhui Cong, Rui Li, Weiqiang Zhang

**Affiliations:** 1 School of Economics and Management, Shanxi University, Taiyuan, 030000 China; 2 China Guangdong Nuclear Engineering Co., Ltd, Shenzhen, 518000 China; 3 School of Business and Economics, Free University of Berlin, 14195 Berlin, Germany

**Keywords:** Energy transition, Labor market, MRIO analysis, Resource dependent area

## Abstract

Achieving carbon peak and carbon neutrality requires not only reducing emissions but also managing the macroeconomic side effects of the energy transition. This study examines employment reallocation as a carbon transition management problem rather than as a conventional labor market performance issue. We develop an integrated multi-regional input-output (MRIO) and multi-objective optimization model to simulate how China’s carbon peak constrained energy transition reshapes formal labor demand across 42 sectors and 31 provinces. The novelty of this study lies in linking a theoretical mechanism of capital biased energy transition with a detailed interregional production network, thereby identifying where the social costs of decarbonization are likely to concentrate. Our results show that although the transition increases aggregate employment, it generates a clear “winner takes all” pattern. Employment gains are captured mainly by economically developed coastal regions and high value added service and power related sectors, while less developed inland and resource dependent regions face severe losses. Shanxi, a typical coal dependent province, could lose nearly 10% of formal employment opportunities, and job growth in renewables is insufficient to fully offset losses in traditional fossil fuel and power industries. These findings provide policy relevant evidence for carbon management by showing that carbon peak pathways must be evaluated not only by emission outcomes but also by their spatial and sectoral employment risks.

## Introduction

The urgent global call for a just energy transition away from fossil fuels to renewable sources was a central theme at COP28. This momentum reflects a growing consensus that a rapid and equitable energy transition is not only critical for climate mitigation but also essential for fostering economic resilience and sustainable development in the face of escalating climate challenges. The core aim of this transition is a fundamental transformation from traditional fossil energy systems to those based on renewables. For China, achieving its carbon peak and carbon neutrality goals will require the widespread deployment of renewable energy and the accelerated phase out of coal after 2025. However, a transition of this scale will inevitably incur substantial shocks to society and the economy, particularly concerning employment. Therefore, maintaining fairness and justice during this process is essential, which requires considering key economic and social goals, such as stable employment and income.

Recent studies published in Carbon Balance and Management have built a closely related knowledge base on China’s carbon management. Yang et al. [1] reviewed China’s national low carbon pathway and emphasized that uniform low carbon policies may be ineffective for regions with different development conditions. Liu et al. [2] used provincial carbon budget scenario simulation to demonstrate substantial spatial heterogeneity in China’s carbon sources, sinks, and peak pathways. Xie et al. [3] examined economic development performance under carbon emission constraints and showed that energy efficiency, urbanization, and scale efficiency shape regional low carbon outcomes. Lin et al. [4] highlighted the need for coordinated control of greenhouse gases and air pollutants, while Zhang et al. [5] and Fan and Wu [6] further showed that carbon peak pathways and energy industry restructuring differ markedly across cities. Collectively, these studies indicate that carbon management is no longer only a problem of estimating emissions or identifying low carbon technologies; it also requires evaluating how transition pathways interact with regional development, industrial restructuring, and social stability. Our study directly extends this agenda by quantifying how a carbon peak constrained energy transition reallocates formal labor demand through China’s interregional production network.

From this carbon management perspective, this paper explores the multifaceted employment impacts of China’s energy transition under the constraint of optimal carbon peak targets. The central concern is not whether the labor market performs well in a general sense, but whether the transition pathway creates spatially concentrated social costs that could weaken the feasibility and fairness of carbon peak management. The answers to the following questions are therefore highly important for China and other developing economies seeking a transition that simultaneously advances emission reduction, economic stability, and social justice:

1. What are the theoretical mechanisms of labor market adjustments underlying the impact of the energy transition on employment?
2. How do the employment effects of the energy transition differ between resource dependent regions and other regions?
3. What is the spatial distribution of the employment effects brought about by the energy transition?

The existing literature on climate change policies and economic growth or labor market, is primarily focusing on three topics. First, the impact of energy transition on the economic growth of developed economies [7]. Second, the impact of climate change policies, such as carbon trading markets and carbon taxes, on the labor market and their complex creation or crowding out effects on employment [8–10]. Third, the effect of technological progress related to energy transition on economic growth and employment at theoretical level [7, 11]. However, few of the previous studies have comprehensively explored the impact of energy transition on the labor market of developing economies and its regional heterogeneity from both theoretical and empirical aspects.

To close the above research gaps, this paper constructed a theoretical framework based on the Cobb-Douglas production function and used the input-output method and multi-objective optimization model to examine the impact of energy transition on employment under the constraints of China’s carbon peak target, thereby providing comprehensive theoretical and empirical evidence. We found that (1) under the constraint of the carbon peak goal, although the energy transition can increase China’s overall labor demand, it leads to a “winner takes all” scenario for regional labor markets. Overall, the labor force migration shows an obvious trend from north to south, from west to east, and from inland to coastal regions. (2) Underdeveloped regions cannot compensate for labor force losses in the short term because the capital energy technology stock has not been adjusted to a balanced state, thus causing labor force migration. Conversely, owing to rapid establishment of capital technology stock and accumulation of large amounts of capital in a short duration of time, economically developed regions accommodate maximum labor force and create new jobs, thus generating greater labor demand. (3) The demand for labor in resource dependent regions declines, resulting in labor migration. In these regions, the rapid adjustment of capital energy technology stock and short term capital accumulation are insufficient to compensate for unemployment caused by energy transition, leading to the phenomenon of labor force flight. (4) The scale of workers in China’s renewable energy industries (primarily wind and photovoltaic power generation industries) will increase to more than four times the current number. However, the employment growth in the renewable energy industries is difficult to make up for the gap in the original coal and power industries.

To address the aforementioned research gaps, this study provides comprehensive theoretical and empirical evidence on the spatial and sectoral employment impacts of China’s energy transition. Its contribution is threefold. First, it reframes employment as a key management dimension of the carbon peak transition. Unlike conventional labor economics studies that focus primarily on wages, participation, or firm level employment behavior, we analyze employment reallocation as a macro level side effect of energy restructuring under carbon constraints. This framing makes the study relevant to carbon management because a technically feasible decarbonization pathway may still be socially fragile if its employment costs are spatially concentrated. Second, the study advances beyond standard MRIO analyses. While conventional MRIO models typically rely on static final demand shocks, our framework couples a detailed MRIO table with a multi-objective optimization setup, allowing us to simulate labor demand adjustments under forward looking carbon peak, energy consumption, and economic growth constraints. Third, the study advances beyond highly aggregated optimization models by preserving interregional supply chain linkages across 1,302 provincial and sectoral units. This granularity allows us to show how the phase out of fossil fuel activities in resource rich provinces can propagate through the national production network and produce a “winner takes all” outcome in which developed regions and high value added sectors capture most employment dividends while traditional resource dependent areas bear disproportionate job losses.

The rest of this paper are organized as follows. Section "Literature review" reviews the literature. Section "Theory framework" describes the theoretical model derivation. Section "Methodology" introduces the research methodologies. Section "Scenario setting" sets the scenario. Section "Empirical findings" analyzes the empirical results, and Sect. "Discussion" presents the research conclusions and policy implications.

## Literature review

This study contributes to the research in two related fields. The first is energy transition and economic growth and the second is policies to address climate change and employment. We commented on previous studies and clarified the research gaps in these two fields.

The impacts of energy transitions on macroeconomic development have been widely discussed in climate change research, particularly in developed countries with rapid transformation progress. The transformation of low carbon energy and power will force the gradual elimination of coal, while driving large scale developments of renewable energy. Changes in production efficiency and energy technology have complex impacts on the economy and society. The transition process of developed economies (such as Europe and the United States) is relatively fast, and they have long been concerned about the possible impacts on the economy and society. Kander and Stern, [7] studied the role of energy substitution in Sweden’s economic growth from 1850 to 1950. A counterfactual simulation was carried out by constructing nested CES production functions and setting exogenous technical assumptions. They believed that the contribution of modern energy to economic growth is far greater than that of traditional energy, however, over time, innovations in labor force technology have gradually become the most important single driving force for economic growth. Qi and Li, [12] used a panel threshold effect model to assess the impact of energy transitions on EU economic growth and found that renewable energy consumption has a negative impact on economic growth. In recent studies, Swilling et al. [13] developed a theoretical framework to examine the impact of the energy transition on economic growth based on a case study of South Africa. They identified several pathways, including the effects of auction mechanisms and the influence of multinational corporations. When renewable energy subsidies are too high, the economic costs become more serious. However, owing to the late start of energy transitions in developing countries, there are few studies on the impact of energy transitions on economic growth and other macroeconomic variables in developing economies.

The Carbon Balance and Management literature is particularly relevant to this study because it has moved from carbon accounting toward policy relevant transition management. For example, provincial carbon budget simulations reveal that China’s carbon peak pathway is strongly shaped by spatial heterogeneity in energy consumption and carbon absorption [2]. Studies on economic development performance under carbon constraints further show that low carbon transition outcomes depend on regional energy efficiency, scale efficiency, and urbanization processes rather than on a single national pathway [3]. Similarly, research on correlated greenhouse gas and air pollutant emissions emphasizes the need for coordinated mitigation policies [4], and recent city level studies show that energy industry structure optimization and carbon peak timing vary across urban development contexts [5, 6]. These studies provide the carbon balance and carbon management foundation for our analysis, but they do not directly quantify the employment risks generated by a carbon peak constrained energy transition. Our study fills this missing social management dimension.

Compared to energy transitions and economic growth, the existing literature predominantly focuses on the relationship between climate change policies and the labor market. Chateau and Saint-Martin, [14] used the Computable General Equilibrium (CGE) model to quantify the impact of OECD countries’ climate change policies on the labor market and employment. It was found that the combination of carbon and labor tax exemptions can minimize the possible negative impacts on the economy, society, and labor market, while emphasizing that a redistribution policy is crucial. Fan et al. [15] used a multiregion CGE model to simulate the impact of China’s unified carbon market on the regional economy and labor market. They found that the establishment of a unified national carbon market can reduce social costs, improve the welfare of workers, and narrow the regional economic development gap. Brown et al. [16] used a CGE model to simulate the impact of implementing carbon tax policy on employment in the United States and found that a carbon tax could significantly increase employment opportunities in the United States, and owing to the path dependence of power investment portfolio and regional resource endowment differences, this effect showed heterogeneity between regions. Godinho, [17] found by combing the literature on the employment effects of climate policies that relevant studies generally believe that the impact on employment is usually moderate, net positive or neutral, but the distribution results may be uneven, which is not conducive to some groups, and sometimes exacerbates the existing inequality. Liu et al. [18] utilized panel data from India spanning 1951 to 2011 and found that rising temperatures significantly reduced employment in the nonagricultural sector, attributing the main causes to the demand side. In the discussion of employment related issues, these studies have paid attention to regional differences in the employment effects of climate policies and used empirical methods for analysis. However, policies to deal with climate change are inseparable from carbon emission reductions. Resource dependent regions may be affected more by these policies. Few studies have focused on the impact of climate policies on employment in resource dependent regions.

Recent studies have focused on the impact of China’s energy transition on the labor market. Hamilton et al. [19] studied the impact of coal transition on regional employment in China’s coal supply chain and found that regions with large total coal employment are often regions with high coal output and consumption, high contribution of the coal industry to GDP, and low coal mining productivity. Therefore, the impact of coal transformation exhibits regional heterogeneity. Sun et al. [20] used a single region input-output methodology to analyze the impact of the power industry transition on China’s labor market and its regional differences. They found that by 2060, low carbon power transformation will have a negative impact on the coal power, coal mining, and washing industries and a positive impact on the machinery manufacturing and equipment industries. Accelerating energy transformation should increase output by 8.21%, employment by 7.97%, and add value by 8.20%. Both studies focused on specific industries in China’s energy transformation and did not involve the analysis of resource dependent regions, and also did not use the multiregion input-output methodology to test regional employment migration. More importantly, existing theoretical research on the relationship between energy transition and the labor market remains quite limited. Füllemann et al. [21] calculated the impact of the energy transition on employment in Switzerland using input-output tables and found that the energy transition overall increased employment, with more jobs gained in demand side industries. Černý et al. [22] developed a multi-regional input-output model to analyze the net employment effects in the power sector by 2050, discovering that the energy transition increased employment in the electricity sector. Following this is a study by Zhang et al. [23], which is highly similar to our research. They used panel data from 2008 to 2022 to analyze the impact of renewable energy transition on employment in China and found that the energy transition boosted employment in the tertiary sector and southeastern regions of China. However, our findings differ significantly from theirs, particularly regarding whether the energy transition benefits low income regions and alleviates inequality. Unlike their conclusions, we argue that China’s energy transition has triggered a “winner takes all” effect at both the sectoral and regional levels, which has exacerbated inequality. We found that no existing studies have used input-output models to simultaneously analyze the regional and sectoral impacts of energy transition on employment, despite the ability of input-output models to provide more detailed insights. Moreover, there is a lack of research addressing the situation in resource rich regions, which are of significant importance. Our study aims to fill this gap by focusing on these critical aspects. Korinek and Stiglitz, [11] believed that the technological progress brought about by energy transition was very similar to artificial intelligence (AI) and constructed a Cobb-Douglas production function for analysis. They found that AI always led to inequalities in global wealth distribution. In contrast to Kander and Stern [7], they believed that this technological progress is endogenous. that is, it will change the substitution elasticity of capital and labor. However, they did not directly study the impact of energy transitions on the labor market but focused on AI.

In summary, previous research deficiencies are threefold: (1) there is still limited evidence on the employment consequences of energy transition in developing economies under explicit carbon peak constraints; (2) few studies connect a theoretical mechanism of capital biased energy transition with an empirical framework capable of tracing interregional production linkages; and (3) existing carbon management research has paid insufficient attention to the spatial concentration of employment risks in resource dependent regions. Our study compensates for these deficiencies by extending carbon peak analysis from emission and economic outcomes to the management of formal labor demand reallocation.

## Theory framework

The essence of labor market adjustment caused by energy policy is that the social planner actively promotes the reallocation of capital and labor factors, a mechanism very similar to that of alternative technological progress. Our analysis starts with a factor price frontier derived from a simple Cobb-Douglas production function and then transitions to a discussion of social welfare. Before beginning, we must specify the following four basic assumptions that underpin our entire analysis.

Persistent wage rigidity: We acknowledge that the assumption of persistent wage rigidity, where the marginal output of labor remains constant, is a stringent simplification for long term analysis in a competitive market. However, this assumption is justified and grounded in China’s specific institutional context and explicit policy objectives. For instance, provincial minimum wages have consistently increased or at least remained stable from 2017 to 2022, even during economic downturns, reflecting a clear policy commitment to a stable income floor. State-owned enterprises (SOEs), which are dominant employers in many resource dependent regions, are also directed by the government to prioritize employment stability over wage reductions. This policy stance is further emphasized in China’s “14th Five-Year Plan,” which mandates robust measures to stabilize employment and ensure social security. Given this institutional backdrop, we argue that the social planner’s commitment to employment and wage stability can effectively limit labor compensation losses in the face of economic shocks. Thus, for our analysis, wages are held constant, a crucial assumption whose implications and limitations are discussed in detail in later chapters. Thus, for our analysis, wages are held constant. Crucially, this wage rigidity explains why our model generates critical threshold values rather than the smooth, continuous labor reallocation seen in standard neoclassical models. In standard models, factor markets clear smoothly because wages fall to absorb shocks. By disabling this smooth price-clearing mechanism to reflect institutional reality, any structural shock that crosses a specific capital labor threshold is forced to manifest as sharp, discontinuous adjustments in labor quantity. This mathematically generates a threshold-driven effect, accurately mirroring the stark “winner-takes-all” polarization observed in our empirical results.

The existence of low cost social redistribution: Based on the above assumption, we argue that, thanks to the long established profit distribution systems of Chinese enterprises and intensified anti corruption measures in recent years, social redistribution can be implemented efficiently at low cost. In other words, the Chinese government will actively suppress rent seeking behavior, ensuring that any excess returns to capital are effectively channeled into investment and employment, rather than exacerbating wealth inequality. While our model assumes frictionless labor mobility, in reality, China’s hukou (household registration) system significantly restricts inter-regional migration, particularly from rural or resource dependent areas to urban centers. Introducing a gradient parameter reflecting these migration barriers would be recommended for future research.

The market is sufficiently competitive in reality: Although achieving perfect competition is impossible, the market closely approximates full competition. We assume that factor inputs at their maximum productive potential are efficient without policy interference.

Factor mobility across regions is adequate: Unhindered cross regional flow of labor factors is crucial for regional analysis.

Before proceeding to the derivations, several critical conceptual definitions and policy mappings must be clarified:

Factor definitions: In our framework, “capital” ($K$) broadly encompasses physical machinery, technological investments, and green infrastructure, explicitly excluding land, as the primary constraint in China’s industrial energy transition is technological rather than spatial. “Resource dependent” refers strictly to regions heavily reliant on traditional fossil fuels, predominantly coal.

Representation of the carbon peak: The “carbon peak” target is not a static quota but is represented theoretically as an exogenous policy shock that simultaneously forces an accelerated reduction in fossil fuel inputs ($N$) and triggers induced technological progress (changes in $\:A$ or $\:\alpha\:$).Redistribution mechanism: When the model refers to social redistribution, it implies a fiscal transfer from the “winners” (entities and regions capturing excess capital returns from green technologies) to the “losers” (displaced workers in traditional fossil fuel sectors).

### Overall employment analysis

We first defined a simple two factor Cobb-Douglas production function.

$$\:F\left(K,L\right)=A{K}^{\alpha\:}{L}^{1-\alpha\:}$$

$$\:L\left(K\right)={\left(\frac{\overline{Y}}{A{K}^{\alpha\:}}\right)}^{\frac{1}{1-\alpha\:}}$$

where $\:K$ refers to the quantity of capital, $\:L$ represents the quantity of labor, $\:\alpha\:$ is the substitution elasticity, $\:A$ is the exogenous technological progress. The capital and labor factor prices are defined as follows:

$$\:\begin{array}{c}{F}_{K}=\frac{\partial\:F(K,L)}{\partial\:K}=r=\alpha\:A(L/K{)}^{1-\alpha\:}\\[2mm] \:{F}_{L}=\frac{\partial\:F(K,L)}{\partial\:L}=w=(1-\alpha\:)A(K/L{)}^{\alpha\:}\end{array}$$

where $\:r$ is the price of the capital factor, that is, the rental price, $\:w$ refers to the price of labor, that is, wages.

**Fig. 1:**
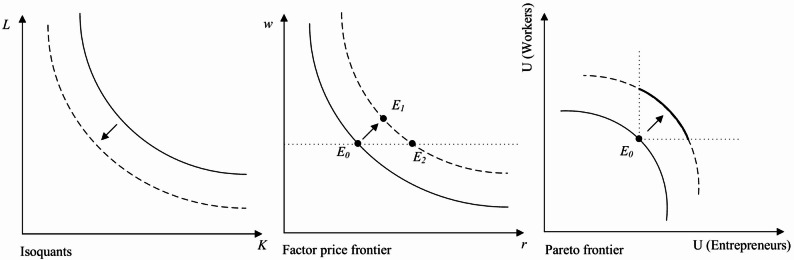
Isoquants, factor price and pareto frontiers

It is assumed that the energy transition under the carbon peak goal will cause a Hicks-neutral impact on technological progress (in fact, such technological progress is usually substitutive, that is, non-Hicks-neutral. Considering that China is not a resource exporting country, we first assumed that this technological progress is Hicks-neutral in general)^1^. In this simple model, when a positive exogenous technological progress impact (i.e., Hicks-neutral technological progress) $\:dA$ is given, an increase in $\:L\left(K\right)$ means that the production possibility frontier moves inward, that is, a greater output can be obtained under the same input. Accordingly, the isoquants curve moves outward (Fig. 1) and the equilibrium point $\:{E}_{0}$ moves to $\:{E}_{1}$. When considering a regional non-Hicks-neutral situation, the equilibrium point will move to the lower right, meaning capital compensation replaces labor compensation. Without policy intervention, the worst case scenario is that labor remuneration would be severely damaged by capital’s rent seeking behavior under the transition shock. However, owing to the existence of China’s structural redistribution policy, we assume these excess capital gains are aggressively taxed or redirected to maintain wage stability (the equilibrium point moves to $\:{E}_{2}$), transforming potential wage collapse into measurable employment quantity adjustments.

Owing to the existence of the redistribution policy, the rent seeking activities of capital will be limited at this stage, and the excess capital gains will be redistributed to balance social welfare. The pareto frontier illustrates the redistribution process. With a constant supply of factors, the utility possibility curve moves outward, owing to the increase in total output and redistribution in a fully competitive market. The solid part of the imaginary arc in the figure represents the possible falling point of a new equilibrium point. It can be seen from this that under the assumption of constant wages, the redistribution policy ensures that the total utility of workers does not decline, meaning that excess rent may create more jobs without damaging workers’ wages through social redistribution and ultimately improve social welfare.

### Regional and sectoral employment analysis

The previous baseline discussion of Hicks-neutral technological progress serves only as a simplified macroeconomic starting point. In reality, the profound regional imbalances in China mean that the transition is highly heterogeneous. Therefore, the regional (rather than “partial”) energy transition is inherently non-Hicks-neutral. It distinctly manifests as energy saving and labor saving technical progress, particularly in resource-rich provinces. Based on this, we discuss regional adjustments below. Based on this, we discuss the transition of alternative energy.

(1) Capital endowment region.

**Fig. 2:**
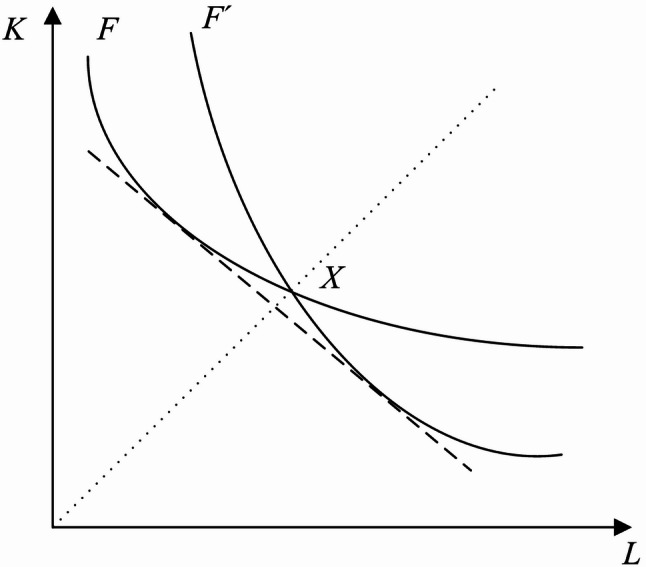
Production possibility frontier under non-Hicks-neutral technology progress

For capital endowment regions, resources are assumed to be exogenous, therefore, we continued to use the dual factor production function. When an alternative technological progress shock $\:d\alpha\:$ is given, the production possibility curve rotates clockwise with the center of the line $\:L=K$^2^ (see Fig. 2). When $\:\frac{K}{L}=\widehat{k}>1$, that is, when the production possibility curve is above the dotted line, the technological progress brought about by the energy transition is efficient, and the market spontaneously promotes the technological progress of energy. We defined a region that meets this situation as the capital endowment region.

In the short term, given a positive shock $\:d\alpha\:$, its impact on the marginal income of the labor force is as follows:

$$\:\frac{d{F}_{L}}{d\alpha\:}={A\widehat{k}}^{\alpha\:}\left[\left(1-\alpha\:\right)ln\widehat{k}-1\right]$$

let $\:\frac{d{F}_{L}}{d\alpha\:}=0$, we conclude that the critical value of capital labor ratio is:

$$\:\stackrel{\sim}{k}\:={e}^{\frac{1}{1-\alpha\:}}$$

when $\:1<k<\:\stackrel{\sim}{k}$, then $\:\frac{d{F}_{L}}{d\alpha\:}<0$, indicating that labor remuneration is negatively impacted. At this stage, if the wage assumption remains unchanged, the redistribution mechanism must be involved, and the expected scale of employment will decline in the short term. When $\:k>\:\stackrel{\sim}{k}$, labor remuneration is impacted positively, the terms of trade in the region are improved, which attracts the inflow of labor, and the expected scale of employment rises. The following equation can be obtained under the assumption that the salary in this study is constant, that is, $\:k=\:\stackrel{\sim}{k}$:

$$\:L={Ke}^{\frac{1}{\alpha\:-1}}$$

it can be seen that when the marginal income share of capital increases, the demand for labor force in the short term will depend on the change in the amount of capital, that is, when the amount of technology investment triggered by the energy transition is sufficient, the demand for labor force can rise, and vice versa. Next, we considered the long term situation.

In the long term analysis, the equilibrium return rate of capital depends on the long term interest rate and depreciation rate of technology, while the long term interest rate depends on the time preference and consumption growth rate. Finally, the interest rate of capital returns to a stable equilibrium level and no longer depends on the proportion of capital and labor in the short term. Referring to the setting of Korinek and Stiglitz [11], we defined the long term equilibrium return rate of capital as follows:

$$\:{F}_{K}^{*}=\rho\:+\delta\:+\mu\:g$$

where $\:\rho\:$ is the time preference, $\:\delta\:$ is the depreciation rate, $\:\mu\:$ is the marginal utility elasticity, $\:g$ is the consumption growth rate. The long term capital stock can be obtained by substituting $\:{F}_{K}^{*}$ as the constant marginal return of capital into $\:F\left(K,L\right)$:

$$\:{K}^{*}={\left(\frac{{F}_{K}^{*}}{\alpha A{L}^{1-\alpha\:}}\right)}^{\frac{1}{\alpha\:-1}}$$

so far, we have obtained the long term production function as follows:

$$\:F\left({K}^{*},L\right)=A{\left(\frac{{F}_{K}^{*}}{\alpha A}\right)}^{\frac{\alpha\:}{\alpha\:-1}}{L}^{\alpha\:(1-\alpha\:)}$$

the impact of the energy transition on the long term marginal income of the labor force becomes

$$\begin{aligned}\:&\frac{d{F}_{L}^{*}}{d\alpha\:}\\ &\quad=-\frac{{\left(\frac{{F}_{K}^{*}}{\alpha\:}\right)}^{\alpha\:/(\alpha\:-1)}{L}^{(\alpha\:-1)\alpha\:}\left[(\alpha\:-1{)}^{2}-\alpha\:\mathrm{l}\mathrm{n}\:\frac{{F}_{K}^{*}}{\alpha A}+\alpha\:(2\alpha\:-1\left)\right(\alpha\:-1{)}^{2}\mathrm{l}\mathrm{n}\:L\right]}{\alpha\:-1}\end{aligned}$$

and the long term critical value of labor quantity becomes:

$$\:{\stackrel{\sim}{L}}^{*}={\left[\frac{\alpha A{e}^{\frac{{(1-\alpha\:)}^{2}}{\alpha\:}}}{{F}_{K}^{*}}\right]}^{\frac{1}{(1-2\alpha\:){(1-\alpha\:)}^{2}}}$$

when $\:L>{\stackrel{\sim}{L}}^{*}$, the long term marginal income of the labor force is positively affected, the terms of trade are improved, and the region will have a siphon effect on the labor force in other regions. When $\:L<{\stackrel{\sim}{L}}^{*}$, the long term marginal income of the labor force is negatively affected, causing a social redistribution mechanism that adjusts the labor market. Under the assumption of constant wages, namely, $\:L={\stackrel{\sim}{L}}^{*}$, given a positive impact, $\:d\alpha\:$, $\:L$ increases. This shows that, in the long run, if wages do not decline, the energy transition will increase the demand for labor in capital intensive regions, which will lead to employment growth.

(2) Labor endowment region.

Corresponding to the previous discussion, in the dual factor production function, we assume that when $\:\frac{K}{L}=\widehat{k}<1$, the region is a labor endowment region. At this time, the proportion of the equilibrium factors falls above the dotted line in Fig. 2. It can be seen that it is more efficient not to carry out technological innovation at this time, that is, for labor endowment regions, the market will not spontaneously promote energy transition. However, energy transformation under the carbon peak is a national policy under the framework of climate change, which means that there is a possibility of resource waste in promoting energy transformation in such regions.

When discussing capital endowment regions, we found that when $\:\frac{K}{L}=\widehat{k}<1$, $\:\frac{d{F}_{L}}{d\alpha\:}$ is always negative, that is, in a fully competitive market, the marginal income of labor is always impaired. Therefore, without intervention, the energy transition will certainly worsen the terms of trade in the region in the short term, reduce labor demand, and lead to labor flight. If wages are stable, a redistribution policy is needed to compensate for wages. We constructed a function $\:A\left(\alpha\:\right)$ about $\:\alpha\:$ to define this kind of compensation. At this time, the critical value of the capital labor ratio becomes

$$\:\stackrel{\sim}{k}\:={e}^{\frac{1-A\left(\alpha \right)}{1-\alpha\:}}$$

it can be seen that, when a redistribution mechanism is involved, the situation of labor endowment regions will become similar to that of capital endowment regions in the short term.

In the long run, we found that the long term marginal income of capital always returns to the equilibrium level. The impact of the energy transition on the long term marginal income of labor is not related to the amount of capital but only to the amount of labor in the base period and the trend and conditions of long term labor demand changes in labor endowment regions are the same as those in capital endowment regions.

(3) Resource endowment regions.

Next, we considered a more complex case. Resource dependent regions refer to regions in which resources are regarded as endogenous growth factors. To some extent, such regions often rely on the export of endowment resources to other regions for growth. Therefore, we reconstructed a three-factor production function that includes traditional energy resources (such as coal):

$$\:Y=F\left(K,L,N\right)=A{K}^{\alpha\:}{N}^{\gamma\:}{L}^{1-\alpha\:-\gamma\:}$$

where $\:\gamma\:$ represents the alternative elasticity of resource element $\:N$. For resource dependent regions, energy transformations are often both labor saving and resource saving technological progress, that is, energy transitions reduce the amount of labor and energy at the same time, but will not reduce the elasticity of resources (assuming that the traditional energy efficiency remains unchanged). Therefore, we provide a positive $\:d\alpha\:$ shock. Because the situation of $\:\widehat{k}<1$ is similar to the previous example, to simplify the analysis, we will only discuss the situation of $\:\widehat{k}>1$, that is, the market will spontaneously promote the energy transition.

Now, the marginal income of the labor force is $\:{F}_{L}=\left(1-\alpha\:-\gamma\:\right)\frac{Y}{L}$, and the short term impact of energy transformation is

$$\:\frac{d{F}_{L}}{d\alpha\:}=\left[\left(1-\alpha\:-\gamma\:\right)ln\frac{K}{L}-1\right]Y$$

the critical value of the proportion of capital to labor is:

$$\:\stackrel{\sim}{k}\:={e}^{\frac{1}{1-\alpha\:-\gamma\:}}$$

therefore, in the short term, the impact of energy transition on labor demand in resource dependent regions is similar to that in other regions. When wages remain unchanged, labor demand also depends on changes in the amount of capital.

However, owing to the introduction of energy and resource elements, the long term results will change. In the long run, when the amount of capital returns to the equilibrium level, the impact of energy transformation on the marginal income of the labor force is as follows:

$$\begin{aligned}\:&\frac{d{F}_{L}^{*}}{d\alpha\:}\\ &\quad=\left\{\frac{1-\alpha\:-\gamma\:}{(1-\alpha\:{)}^{2}}\left[\mathrm{l}\mathrm{n}\frac{\alpha\:A}{{F}_{K}^{*}}+\gamma\:\mathrm{l}\mathrm{n}\frac{N}{L}-\frac{\gamma\:(1-\alpha\:)}{1-\alpha\:-\gamma\:}\right]\right\}{Y}^{*}\end{aligned}$$

let $\:\frac{d{F}_{L}^{*}}{d\alpha\:}=0$, and we found that the critical value is closely related to the ratio of resources to labor $\:\frac{N}{L}$, namely $\:{\stackrel{\sim}{n}}^{*}$, which is defined as:

$$\:{\stackrel{\sim}{n}}^{*}={\left[\frac{\left({F}_{K}^{*}\right)\cdot\:{e}^{\frac{\gamma\:(1-\alpha)}{1-\alpha\:-\gamma\:}}}{\alpha A}\right]}^{\frac{1}{\gamma\:}}$$

when$\:\:\frac{N}{L}<{\stackrel{\sim}{n}}^{*}$, labor wages are damaged, and terms of trade deteriorate, leading to labor emigration. When $\:\frac{N}{L}>{\stackrel{\sim}{n}}^{*}$, labor wage benefits and terms of trade improve, causing the labor siphon effect. When$\:\:\frac{N}{L}={\stackrel{\sim}{n}}^{*}$, that is, wages are unchanged, labor demand is as follows:

$$\:{\stackrel{\sim}{L}}^{*}=N{\left[\frac{\left({F}_{K}^{*}\right)\cdot\:{e}^{\frac{\gamma\:(1-\alpha)}{1-\alpha\:-\gamma\:}}}{\alpha A}\right]}^{-\frac{1}{\gamma\:}}$$

it can be seen that when the elasticity of technology substitution increases and the quantity of resources decreases owing to the energy transition, the demand for labor will certainly decrease. Thus, if wages remain unchanged, resource dependent regions will certainly face shrinking employment in the long run.

In conclusion, this study comprehensively analyzed the impact of carbon peak and energy transition on the short term and long term adjustment of the labor market and draws a conclusion on heterogeneous regions based on factor endowment. The above analysis involves many assumptions and inferences, and a grasp of China’s actual problems still needs the support of empirical analysis. In the following study, we used the input-output method to simulate the static results of labor market adjustment under the carbon peak target. Combined with the theoretical model, this study provides a dynamic conclusion on China’s labor market.

## Methodology

It is crucial to clarify the exact methodological relationship between the theoretical model (Sect. "Theory framework") and the empirical simulation (Sect. "Methodology"). They are not mathematically coupled; rather, they serve distinct but complementary analytical purposes. The theoretical model provides the microeconomic foundations (the “why”), demonstrating how capital biased technological progress inherently squeezes out labor when crossing specific thresholds. However, theory alone cannot quantify the magnitude or spatial distribution of these structural shocks. Therefore, the MRIO model serves as the macro empirical quantification tool (the “what” and “where”), tracing how these theoretically predicted factor substitutions physically propagate through China’s complex interprovincial supply chains.

### Overview of the modelling pipeline

To enhance transparency and provide a clear roadmap of our analytical framework before delving into the mathematical equations, we outline the modelling pipeline in four sequential steps:

Step 1: Inputs and MRIO construction. We utilize the 2017 detailed multi-regional input-output (MRIO) table covering 42 sectors across 31 Chinese provinces (1,302 provincial and sectoral units). This serves as the baseline economic structure, capturing the complex, interconnected interregional and intersectoral supply chain linkages.

Step 2: Optimization setup. We formulate a multi-objective optimization model built upon the MRIO framework. The objective function is designed to balance three primary macro goals simultaneously: maximizing economic growth (GDP), minimizing total carbon emissions, and minimizing total energy consumption.

Step 3: Solution procedure under scenario constraints. We apply a set of forward looking policy and technical constraints, such as GDP growth bounds, carbon peak targets, industrial adjustment limits, and structural shifts driven by electrification and efficient coal utilization [24], across eight distinct development scenarios up to 2035. The model is then solved to identify the optimal trajectory (subsequently identified as Scenario 6) that balances emission reductions with economic stability.

Step 4: Mapping results to regional and sectoral employment. Finally, the optimized economic outputs (value-added per sector and region) from the chosen scenario are mapped back to employment figures. We use specific labor productivity coefficients, dynamically adjusted for future demographic and structural trends, to translate these projected economic shifts into granular job creation or displacement across all 1,302 units.

The following subsections detail the specific mathematical formulations and data processing techniques used in these steps.

### Input-output multi-objective optimization model

Before combining with the employment data, it is necessary to measure future growth under the constraint of carbon neutral targets. The input-output multi-objective optimization model uses input-output data and sets reasonable objective constraint functions to achieve simulation predictions to determine the optimal carbon peak scenario. This model establishes a multi-objective optimization model based on the scenario analysis method and the 2017 input-output table, maximizes economic development, minimizes carbon emissions, and minimizes energy consumption as the three development goals of economy, carbon emissions, and energy. It analyzes and explores China’s carbon peak scenarios from the perspective of economic development level, peak time, and peak value of carbon emissions, and finds the optimal scenario.

The objective function is as follows:

(1) Economic maximization goals

1 $$\:\begin{array}{c}MaxG=\sum\limits_{i=1}^{n}{v}_{i}, \left(i=1, 2, \dots\:\dots\:, 1302\right)\end{array}$$

(2) Carbon emission minimization targets

2 $$\:\begin{array}{c}M\in\:C=\sum\limits_{i=1}^{n}{{c}_{i}v}_{i},\left(i=1,2,\dots\:\dots\:,1302\right)\end{array}$$

(3) Energy consumption minimization targets

3 $$\:\begin{array}{c}M\in\:E=\sum\limits_{i=1}^{n}{{e}_{i}v}_{i},\left(i=1,2,\dots\:\dots\:,1302\right)\end{array}$$

where $\:G$ is GDP in the target year, $\:{v}_{i}$ is the added value of each department in the target year, and $\:n$ is the number of departments. There were 42 departments in 31 provinces, totaling 1302 departments. $\:C$ is the total carbon emissions in the target year, and $\:{c}_{i}$ is the carbon emission intensity of each department, which was calculated by dividing the carbon emissions of each department in 2017 by the increase in each department. $\:E$ is the total energy consumption in the target year and $\:{e}_{i}$ is the energy consumption intensity of each department, which was calculated by dividing the carbon emissions of each department in 2017 by the increase in each department.

The constraints are as follows.

(1) Economic growth constraints.

Both energy conservation and emissions reduction will constrain economic development to a certain extent. To maintain economic growth, the average annual growth rate of GDP is constrained to be no less than $\:\lambda\:$.

4 $$\:\begin{array}{c}G=\sum\limits_{i=1}^{n}{v}_{i}\end{array}$$

5 $$\:\begin{array}{c}G\ge\:{\left(1+\lambda\right)}^{t}{G}_{0}\end{array}$$

where $\:{G}_{0}$ is the GDP of the base year, $\:t$ is the number of years between the base year and target year, $\:\lambda\:$ is an exogenous parameter.

(2) Constraints on growth in energy consumption.

Currently, there is no decoupling between economic development and energy consumption. To control energy consumption and improve energy utilization efficiency, it is necessary to restrict the growth of energy consumption to not exceed $\:{\mu}_{1}$.

6 $$\:\begin{array}{c}E={\sum\:}_{i=1}^{n}{{e}_{i}v}_{i}\end{array}$$

7 $$\:\begin{array}{c}E\le\:{\left(1+{\mu}_{1}\right)}^{t}{E}_{0}\end{array}$$

(3) Constraints on carbon emission growth.

To achieve the CO2 control target, the growth rate of CO2 emissions should not exceed $\:{\mu\:}_{2}$.

8 $$\:\begin{array}{c}C=\sum\limits_{i=1}^{n}{{c}_{i}v}_{i}\end{array}$$

9 $$\:\begin{array}{c}C\le\:{\left(1+{\mu}_{2}\right)}^{t}{C}_{0}\end{array}$$

(4) Coal reduction constraint

10 $$\:\begin{array}{c}{\sum}_{Coal\:sectors}{v}_{t}\le\:(1-\partial\:){\sum}_{Coal\:sectors}{v}_{t-1}\end{array}$$

In order to achieve the goal of reducing the use of coal, the annual average reduction rate of the coal sector should not be less than $\:\partial\:$.

(5) Constraints on industrial adjustment range.

To avoid excessive economic fluctuations, the industrial scale of the input-output table is limited to change within a certain range, so the constraints of industrial expansion are as follows:

11 $$\:\begin{array}{c}{p}_{1}{v}_{t-1}\le\:{v}_{t}\le\:{{p}_{2}v}_{t-1}\end{array}$$

(6) Nonnegative constraint:

12 $$\:\begin{array}{c}{v}_{i}\ge\:0\end{array}$$

### Calculation of employment

At present, China conducts annual sample surveys on population changes, statistics on urban unit employees, and statistics on rural employees. However, these employment statistics differ in terms of statistical objects, accounting methods, industry classifications, etc., resulting in insufficient employment statistics at the industry level in China. Owing to the lack of uniformity of methods and standards, the comparability of employment data at the industry level in each year is poor [25, 26]. For this reason, some scholars measure the number of industrial employees by the number of urban unit employees [25, 27], while others obtain employment by dividing the employee’s remuneration in the input-output table by the average wage or measure employment opportunities by urban unit employment per unit added value to obtain employment in various industries [28]. However, such methods lack consideration of population growth, which could lead to errors in the results and even to the total number of employed people exceeding the total labor force. Therefore, when using urban unit employees of various industries to calculate the total scale of employment in the industry, we should combine the total scale of employment in the year, the population budget, and other data to make the measurement of the number of jobs closer to the predictions for 2035. The specific processing methods are as follows.

First, according to the added value of each department in the country in 2017 and the number of urban unit employees in each department, the coefficient of the scale of employment in each department’s added value in 2017 was calculated, and the scale of employment in each region and industry in 2017 was calculated. This was used for comparison with the 2035 value under the unified standard. Then, according to the added value of each sector and region under the peak scenario and the employment number coefficient of the unit added value in 2017, the sector regional employment structure in 2035 was obtained, and the actual number of workers was calculated. Finally, the scale of employment in the renewable energy industry was calculated according to the added value of the power industry, the proportion of renewable energy power generation, and the input of various sectors involved in the renewable energy industry.

To ensure a precise interpretation of our main results, it is necessary to clarify what our employment measure represents. The reported employment numbers reflect the formal demand for labor, specifically urban unit employment opportunities. These figures capture both the net changes in total job creation and the extensive structural reallocation of labor across different sectors and regions. Furthermore, certain limitations must be acknowledged. Readers should not infer absolute unemployment rates from these figures, as our model focuses on labor demand and does not capture demographic labor supply dynamics. Additionally, the measure does not account for informal sector employment, nor does it reflect potential fluctuations in actual wage levels, given the wage rigidity assumption applied in our simulations. To further clarify the nature of labor mobility in our framework, it is important to emphasize that our model calculates the spatial and sectoral reallocation of labor demand, meaning job opportunities, rather than simulating the physical cross provincial migration of workers. We assume that labor is relatively mobile across different sectors within the same province. However, cross provincial physical migration is subject to real world frictions such as the household registration (Hukou) system and relocation costs. Thus, when we report job losses in an inland province and gains in a coastal province, it reflects a macro structural shift in economic demand rather than the direct transfer of the same individual workers across regions.

## Scenario setting

Based on historical data and development trends in China’s economic development, energy transformation, and industrial structure adjustment rate, this study sets the change rate of five factors: GDP growth rate, annual carbon emission growth rate, energy consumption growth rate, and the upper and lower limits of annual industrial structure adjustment. The specific setting is as follows:

1. Basis for GDP setting: During the “13th Five Year Plan” period, China’s GDP growth in the first four years was 6.7%, 6.9%, 6.7%, and 6%, with an average annual growth of 6.6%. In 2020, owing to the impact of the COVID-19 epidemic, it was only 2.3%. In the “14th Five Year Plan”, China did not directly propose the GDP development goal, but in 2021, China achieved a sharp increase of 8.1%. Some scholars believe that the effect of the pandemic on GDP growth rate during the “Fourteenth Five Year Plan” period in China was approximately 5.3% − 6.6% [29, 30].
2. Basis for setting the carbon emission growth rate: According to the data of the CEADS carbon emission database, China’s carbon emissions have slowly increased from 8 billion tons to 10 billion tons in the past decade, and the growth rate during the “13th Five Year Plan” period is approximately 3%. In the “14th Five Year Plan”, it is proposed that the carbon emission intensity should be reduced to about 18% in five years, that is, the average annual reduction rate of carbon emission intensity is about 3.36%. In 2021, the growth rate of national carbon emissions is expected to reach 3.71%. Therefore, the growth rate of carbon emissions was set at 3.2% − 4.5%. The solid line represents carbon emissions and the dotted line represents GDP growth (the same below).
3. Growth rate of energy consumption: In 2019, the total energy consumption of the entire year was 4.86 billion tons of standard coal, an increase of 3.3% over the previous year. The “14th Five-Year Plan” Comprehensive Work Plan for Energy Conservation and Emission Reduction proposes that the total energy consumption target be adjusted accordingly for regions whose economic growth exceeds the expected target [31]. Therefore, the average annual growth rate of energy consumption was set at 3% to 4%.
4. Growth rate of coal use: In the past five years, coal output and consumption have tended to be stable, with the growth rate maintained at −3% to 1.5%. At COP26 of the UN Climate Conference, China promised to gradually reduce coal use in the future. The reduction in coal use and the irreplaceability of coal is considered to be chemical raw materials. To achieve the reduction plan, the coal growth constraint was limited to − 3% here.
5. The upper and lower limits of annual industrial added value adjustment. A study of Zheng et al. [28] on Beijing’s industrial adjustment from 2001 to 2010 proposed that the upper and lower limits of the ten years were about (−50% and 150%), that is, the average annual industrial value-added adjustment rate was set at about (−0.06% to 0.1%). The research scope covered 18 years. It is necessary to ensure that the industrial structure adjustment is sufficient to affect GDP, carbon emissions, and energy consumption and to ensure that the model is stable. Therefore, the annual industrial structure adjustment rate was set, as shown in Table 1. Our current model does not explicitly incorporate interactions with China’s national Emissions Trading System (ETS), potentially overestimating employment losses in resource dependent regions. Subsidies or support policies linked to the ETS, such as coal to renewable labor transition support schemes, could mitigate regional unemployment impacts.

As there are many parameters and the research period covers the three periods of the “14th Five Year Plan,” “15th Five Year Plan” and “16th Five Year Plan,” the scenario combination is complex, so the following criteria are proposed to reduce meaningless scenarios:

- According to the objective law, a low industrial structure adjustment rate, low energy consumption, and low carbon emissions can only correspond to medium and low GDP.
- Too high or too low energy transformation intensity will result in a suboptimal path of high carbon emissions and low economy, respectively, so we do not conduct too much scenario research on the two extreme transformation schemes.
- 2017–2025 is still some time away from the carbon peak task period, and the industrial structure adjustment rate considers the high and low development rates.
- 2025–2030 is a critical period for the completion of the carbon peak task and the industrial structure adjustment rate is high.
- After 2030, to avoid drastic industrial replacement, the scenario design of industrial structure adjustment should focus on a low adjustment rate.
- Considering the goal of coal reduction and phase out, the coal growth rate is less than 3%.

The scenario combination in Table 2 was formed according to the scenario parameter setting, objective economic laws, and the above criteria in the previous section.

To facilitate navigation and provide a clear overview of our experimental design, Table 3 presents a comprehensive summary of all eight scenarios. This reference table consolidates the main constraints (economic growth, energy consumption, and carbon emission targets), the time horizon evaluated (2017 to 2035), and the core outputs emphasized in our subsequent empirical analysis, including the projected peak year and macroeconomic indicators by 2035.

Although the carbon peak constraints and economic targets are implemented as national macroscopic parameters in our optimization function, the resulting impacts are inherently province specific. This regional heterogeneity is endogenously driven by the distinct technological coefficients and initial resource endowments of the 31 provinces captured in the multi-regional input-output matrix. For example, a uniform national carbon reduction trajectory naturally imposes much stricter structural adjustment pressures on resource heavy provinces like Shanxi compared to service oriented provinces like Beijing.

## Empirical findings

The empirical analyses of this study are as follows. First, perform parameter simulation according to the established carbon peak scenario and comprehensively determine the optimal peak scenario. Second, under the optimal peak scenario, the change in employment quantity was calculated according to the added value of 42 departments (1302 departments in total) in 31 provinces and cities of China, and the analysis was based on the changes in employment quantity and structure in different provinces, cities, and departments.

### Analysis of energy transition in China and major resource dependent regions under various scenarios

Before discussing the employment effect, we will first carefully determine an optimal carbon peak scenario that is in line with China’s actual situation. The parameters for the optimal scenario will be set according to the requirements of China’s “13th Five Year Plan”, “14th Five Year Plan”, and relevant policy goals for carbon peaking. Since Shanxi Province was established as China’s national energy base in 1980, coal mining, washing and other industries surrounding coal have once become the main pillar of economic development in this region. In 2005, the added value of coal, metallurgy, coke and electric power accounted for 80% of the province’s industrial added value for many consecutive years, and the comprehensive energy consumption per unit GDP was 2.6 times the national average. Therefore, we select Shanxi Province of China as a typical coal resource dependent region for analysis.

(1) Analysis of changes in the economic aggregate and carbon emissions under various scenarios.

**Fig. 3:**
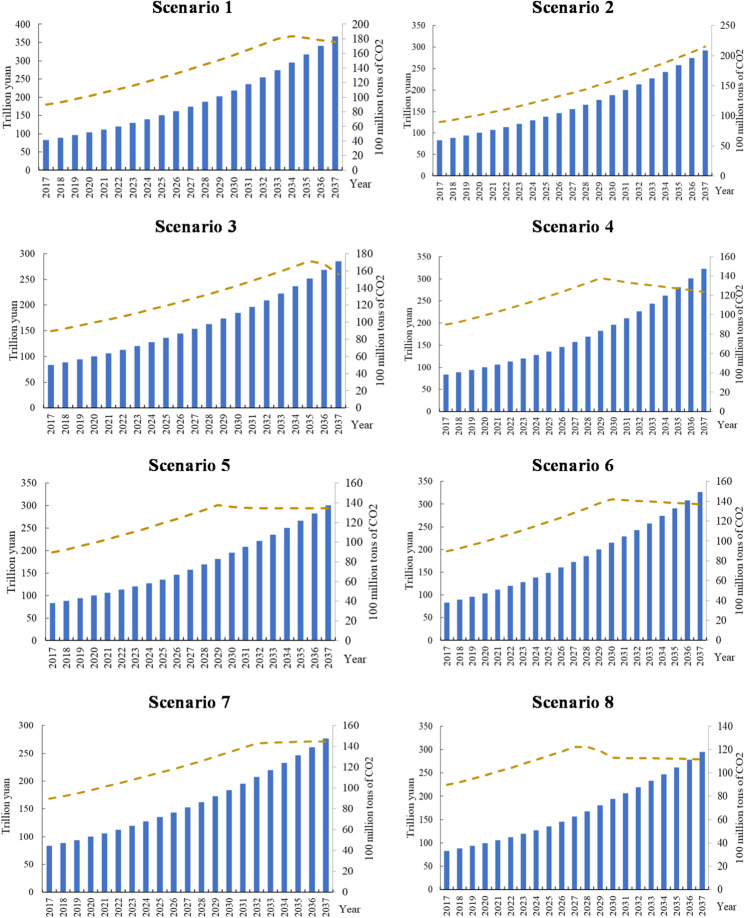
China’s GDP and carbon emission trend under various scenarios. This Figure reports the simulation path of China’s GDP and carbon emissions under various carbon peak scenarios. The column represents GDP, while the line represents carbon dioxide emissions. In order to save space, the data of Shanxi Province was not reported on the figure, and instead we provided those in the text

Based on the optimization model and exogenous parameters, the development path of China in different scenarios from to 2017–2035 was obtained. This study selected the Shanxi Province as a typical resource dependent region for comparative analysis (see Fig. 3).

**Scenario 1**

In Scenario 1, China will reach a peak of 18.389 billion tons of carbon emissions by 2034. In 2035, the total GDP will be 317.37 trillion yuan, and carbon emissions will be 18.097 billion tons. Under this scenario, Shanxi Province will reach its peak of 853 million tons in 2034. In 2035, Shanxi’s GDP will be 5.11 trillion yuan and its carbon emissions will be 825 million tons.

**Scenario 2**

In Scenario 2, the carbon peak will not be achieved by 2035, and carbon emissions will be 19.68 billion tons in 2035. In 2035, the total GDP will be 257.76 trillion yuan and carbon emissions will be 19.68 billion tons. Under this scenario, Shanxi Province also did not reach its peak. In 2035, its GDP will be 4.26 trillion yuan and its carbon emissions will be 781 million tons.

**Scenario 3**

In Scenario 3, China will achieve a peak of 17.137 billion tons of carbon emissions by 2035. In 2035, the total GDP will be 252.13 trillion yuan, and carbon emissions will be 17.137 billion tons. Shanxi Province has not yet reached its peak. In 2035, the GDP will be 4.17 trillion yuan and the carbon emissions will be 775 million tons.

**Scenario 4**

In Scenario 4, China achieves a carbon peak of 13.78 billion tons in 2029. In 2035, the total GDP will be 280.73 trillion yuan, and the carbon emissions will be 12.704 billion tons. Shanxi is expected to reach its peak in 2030, with a peak of 656 million tons. In 2035, the total GDP of Shanxi Province will be 4.58 trillion yuan, and the carbon emissions will be 587 million tons.

**Scenario 5**

The parameters of Scenarios 5 and 4 are the same before 2030, thus, China will also achieve a carbon peak of 13.78 billion tons in 2029. In 2035, the total GDP will be 266.40 trillion yuan, and carbon emissions will be 13.453 billion tons. Shanxi is expected to reach its peak in 2030, with a peak of 656 million tons. In 2035, the total GDP of Shanxi Province will be 4.33 trillion yuan, and the carbon emissions will be 617 million tons.

**Scenario 6**

In Scenario 6, China achieves a carbon peak of 14.19 billion tons by 2030. In 2035, the total GDP will be 290.33 trillion yuan, and the carbon emissions will reach 13.833 billion tons. Shanxi is expected to reach its peak in 2030, with a peak of 693 million tons. In 2035, the total GDP of Shanxi Province will be 4.77 trillion yuan, and carbon emissions will reach 652 million tons.

**Scenario 7**

In Scenario 7, China will achieve a carbon peak of 14.293 billion tons by 2032. In 2035, the total GDP will be 248.44 trillion yuan, and carbon emissions will be 14.051 billion tons. Shanxi will reach its peak in 2032, with a peak at 685 million tons. In 2035, the total GDP of Shanxi Province will be 4.11 trillion yuan, and carbon emissions will be 659 million tons.

**Scenario 8**

In Scenario 8, China is expected to achieve a carbon peak of 12.293 billion tons by 2028. In 2035, the total GDP will be 262.06 trillion yuan, and carbon emissions will be 11.217 billion tons. Shanxi is expected to reach its peak in 2029, with a peak of 630 million tons. In 2035, the total GDP of Shanxi Province will be 4.28 trillion yuan, and carbon emissions will be 475 million tons.

Additionally, based on the comparison of various scenarios, it was found that:1) the smaller the energy transformation, the higher the carbon emissions and total energy consumption, the harder it is to achieve the carbon peak goal, the hidden dangers to achieve the carbon neutral goal will be buried, and the economic and social costs may eventually be higher, the greater the energy transformation, the slower the economic growth and the lower the economic aggregate. He et al. [32] also found that reaching the summit prematurely has a significant inhibiting effect on economic development, and may even affect the realization of the goal of socialist modernization in 2035. 2) A larger adjustment of industrial structure can significantly reduce total carbon emissions and energy consumption, improve economic growth speed, and advance the time of carbon peak, but it has a great impact on industrial development, and some industries fluctuate greatly. Therefore, relevant policies should be carefully implemented. 3) China’s carbon peak is likely to be between 13 billion tons and 14 billion tons, and the GDP in 2035 will be approximately 3–4 times that in 2017. At present, there are different views on the peak carbon timing in China, with some scholars believing that it may occur between 10 and 12 billion tons (Lu and Chen 2021). However, others believe that the peak may be more than 12 billion tons, for example, Yan Gang predicted that the peak may be between 12 billion tons and 122 billion tons (Yan et al. 2022). Under the analysis of low carbon scenarios, Xu et al. [33] proposed that China’s carbon peak is about 14.3 billion tons. Qiao and Peng (2022) believes that China’s carbon peak will be between 13.235 billion tons and 16.615 billion tons. The peak value estimated here is within a reasonable range.

(2) Determination of the optimal peak scenario.

From the perspective of economic cost, China’s GDP under Scenario 1 was the highest at 317.37 trillion yuan, and China’s GDP under Scenario 8 was the lowest at 168.03 trillion yuan. The opportunity cost of vigorously implementing energy transformation, energy conservation, and emission reduction may exceed one million billion yuan per year. From the perspective of carbon emissions, the maximum difference between the peaks is approximately 7 billion tons (the highest is 19.6 billion tons, the lowest is 12.2 billion tons). In other words, different transformation efforts will significantly affect carbon emissions, and the country has the potential to reduce 7 billion tons of CO2 (Table 4). Considering the opportunity cost and carbon emission reduction factors, Scenario 6 was selected as the optimal development scenario, which can consider the constraints of economic growth, carbon peak, and carbon low peak.

### China’s overall employment effect

(1) Calculation of the scale of employment in various departments.

According to the calculation, the scale of employment in each department in 2035 is shown in Table 5. In terms of total number, seven departments realized the growth of urban unit employment, with an increase of 49.8852 million people and 35 departments decreased, with a total decrease of −42.3372 million people and a net increase of 7.5481 million people. In 2017, the sectors with the largest number of urban unit employment in each sector were construction, education, public management, social security and social organization, health and social work, transportation, warehousing, and postal services. In 2035, the sectors with the largest number of jobs should be transportation, warehousing and postal services, power and heat production and supply, leasing and business services, construction, and education, with the largest increase in employment in transportation, warehousing and postal services, production and supply of electricity and heat, leasing and business services, real estate, wholesale and retail. Buildings, non-metallic mineral products, coal mining and dressing products, metal smelting and calendering products, and chemical products are the most reduced. The employment of transportation, warehousing, and postal services and the production and supply of electricity and heat increased significantly. Conversely, the two sectors have a relatively high capacity for employment creation, and the number of jobs created per unit of added value is the fifth and first, respectively. However, in the peak scenario, the above two sectors have been developed to a certain extent, and the added value increases by approximately three and 1.7 times respectively, so the scale of employment increases the most.

Under the constraints of economic growth, carbon emissions, and energy consumption, the development space for buildings, non-metallic mineral products, coal mining and dressing products, metal smelting and calendering products, and chemical products are compressed, which is equivalent to saving carbon emissions and energy consumption space to develop sectors with higher economic benefits. Under the constraint of the carbon peak, the increase in employment in 2035 means that social welfare will increase under the assumption of constant wages, which is consistent with the inference given in the theoretical analysis.

**Fig. 4:**
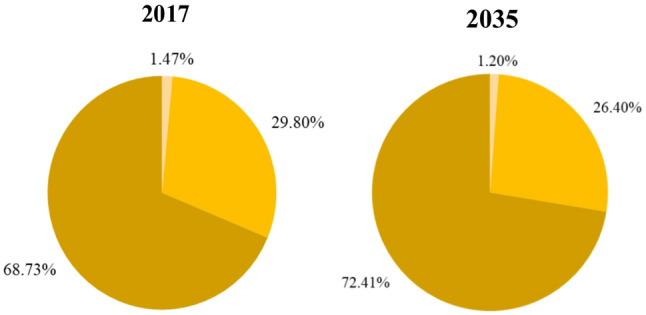
The employment structure of three industries in 2017 & 2035

The scale of employment in the secondary industry will decline by nearly 3.4%, corresponding to an increase of approximately 4% in the number of employed people in the tertiary industry (Fig. 4). The proportion of employment in the primary industry changed slightly and the employment structure was relatively stable. The employment scale of the primary industry, employment scale of the secondary industry, and employment scale of the tertiary industry are shown in the figure from shallow to deep.

From the perspective of sectors, we can see that: first, the employment risk problem is prominent under the transition and development of energy conservation and emission reduction. Most sectors face the risk of employment reduction, and only a few sectors can achieve growth. Second, the tertiary industry plays a significant role in absorbing employment, especially in the logistics, education, intermediaries, and other related industries. At the same time, the economic growth of these industries is among the fastest. Therefore, vigorously developing industries with low emissions, low energy consumption, and high economic benefits, and guiding the unemployed in the secondary industry to reemployment in the tertiary industry will be an important way to deal with employment fluctuations in the future. The optimization of industrial and employment structures should go hand in hand. Third, employment in the power industry increased significantly. In combination with the alternative development path of renewable energy and the development path of electrification, the demand for high tech talents related to electricity will increase dramatically. Therefore, vigorously developing industries with low emissions, low energy consumption, and high economic benefits, and guiding the unemployed in the secondary industry to reemployment in the tertiary industry, will be vital for managing future employment fluctuations. Moreover, the transition policies can motivate possible green technology innovation, which is expected to further expand job opportunities in related high tech and service sectors [34].

(2) Changes in employment by province.

Eighteen provinces experienced employment growth and 13 provinces experienced employment reduction (Table 6). In 2017, Guangdong, Jiangsu, Shandong, Zhejiang, and Henan had the largest employment scales. In 2035, Guangdong, Jiangsu, Shandong, Zhejiang, and Henan will still have the largest scale of employment, but the scale of employment will change by 20,920, 131.38, 665, 6283, and 376,600, respectively. The largest increases in employment were in Guangdong (2.092 million), Jiangsu (1.3138 million), Beijing (775900), Fujian (717600), and Zhejiang (628300), with the largest decreases being in Guizhou (−680400), Sichuan (−631900), Yunnan (−556600), Hubei (−469100), Henan (−376600), and Shanxi (−304700).

The scale of employment in regions with developed economies, low carbon emission intensity, and low energy consumption intensity showed an upward trend, whereas the scale of employment in regions with developed industries or regions with high carbon emissions and energy consumption decreased significantly. Yunnan, Guizhou, Sichuan, and Hubei were mainly affected by the reduction in employment in the construction and real estate sectors. Henan and Shanxi are mainly affected by the coal mining and mineral processing industries. However, despite a significant decline in employment in Henan, total employment still ranks among the top five in China. The labor force transfer shows an obvious trend from north to south, west to east, and inland to coastal areas.

In our previous theoretical analysis, we found that the long term labor force in both developed and underdeveloped areas showed an upward trend under the assumption of constant wages. The empirical results show that the energy transition has reduced labor demand in the underdeveloped regions, indicating that in the underdeveloped regions characterized by labor endowment, the technological progress brought by the energy transition in the capital market has not reached the equilibrium level in 2035, and the labor market has not been fully adjusted. Moreover, in the case of the redistribution mechanism, the technology investment in underdeveloped regions is insufficient to compensate for the crowding out of the labor force caused by the energy transition itself. The rising demand for labor in developed regions indicates that their capital markets have been adjusted to a balanced state or that the transformation has brought intensive technology investment so that they can quickly absorb the displaced labor force and create more jobs in a short period of time.

To succinctly illustrate the “winner takes all” dynamic discussed above, Table X provides a compact ranked summary of the most significantly affected sectors and regions under the optimal carbon peak scenario. This highlights the stark distributional consequences of the energy transition, clearly delineating where job creation is concentrated and where the employment risks are most severe.

(3) Changes in renewable energy industry.

The proportion of renewable energy power generation is expected to be between 35% and 45% by 2035 (Wu et al. [26]). Therefore, the employment number of major renewable energy power generation industries when the proportion of renewable energy power generation is 35% to 45% in 2035 (Table 8). According to the data released by the Global Wind Energy Council (GWEC), there will be about 550,000 people employed in the wind power industry in China in 2020, which is close to the estimate in this study. According to the calculation results, the number of renewable energy sources in the future will increase to four times the current number, and is mainly concentrated in wind power generation and photovoltaic power generation. The growth of employment in the renewable energy industry can alleviate the employment impact caused by transformation and development to a certain extent, but it is far from filling the employment gap.

### Employment effect in resource dependent regions: a case of Shanxi Province, China

(1) Change in employment by sector.

The most employed sectors in 2017 were the production and supply of electricity and heat, transportation, warehousing and postal services, coal mining and processing products, public management, social security and social organizations, and education, of which the number of coal mining and processing employees ranked third (Table 9). The sectors with the most employment in 2035 are the production and supply of electricity and heat, transportation, warehousing and postal services, public management, social security and social organizations, education and construction. The sectors with the largest increase in employment are transportation, warehousing and postal services, public administration, social security and social organizations, education, construction and real estate. In 2035, the sectors with the greatest job losses will be coal mining and processing products (419,600 people), power and heat production and supply (263,500 people), metal smelting and calendering products (66,800 people), non-metallic mineral products (20,500 people), petroleum, coking products and nuclear fuel processing products (13,900 people).

The number of urban units employed in Shanxi Province has decreased by nearly 10%, and the scale of employment in the coal mining and dressing industry and the production and supply departments of electricity and heat has decreased by 85% and 25%, respectively, mainly due to coal constraints. In the case of phasing out the coal industry, combined with population growth factors, the employment reduction rate in the coal industry will exceed the shrinking rate of industrial added value. However, if the power industry can improve the status quo of coal fired power generation, its actual development may be better than that of the scenario. Owing to the slow growth of employment in other sectors, the scale of employment in the power sector is still higher in the province. It can be seen that the transformation and development of Shanxi Province under the goal of reaching the carbon peak will have a great impact on the scale of employment in the coal and power sectors, and the reduced scale of employment will make it difficult to achieve balance through employment accommodation in other industries in the province, which will lead to a decline in the labor force and transregional transfer. The reported 10% employment loss in Shanxi should be viewed with sector specific context. Specifically, the employment reduction trajectories differ significantly between coal mining (approximately − 85%) and coal power plants (approximately − 25%). Future research should align these detailed employment trajectories with the NDRC’s coal power flexibility transition guidelines for clearer policy implications.

In terms of the total amount, by 2035, energy transformations under the carbon peak goal could reduce employment in China’s resource dependent regions and reduce the demand for labor. Based on the above theoretical analysis of this type of region, it can be seen that the capital market of the resource dependent region has been adjusted to a balanced state, or there is not enough technology investment in the short term to compensate for the labor loss caused by energy transformation.

(2) Renewable energy employment.

The proportion of renewable energy power generation in Shanxi Province is expected to be approximately 25% to 35% by 2035 [35]. The number of jobs in each major renewable energy power generation industry when the proportion of renewable energy power generation will be 25% to 35% in 2035 (Table 10). The number of people in all types of energy showed an upward trend, reaching 150,000 in total. However, employment growth in the renewable energy industry may not compensate for the employment gap in the original coal and power industries, and employment problems in the future are still very possible.

## Discussion

This study constructs a theoretical analysis framework based on a production function to examine the impact of energy transition on employment. Building on this, we developed a multi-regional input-output model to conduct detailed empirical simulations from both regional and industry perspectives. Our findings reveal a “winner takes all” effect of the energy transition in China at both industry and regional levels: sectors and regions that were already performing well gain more benefits, while less developed regions and industries face negative impacts. Furthermore, the energy transition significantly reduces employment in resource dependent regions, especially in industries related to traditional fossil fuels, and this job loss is unlikely to be fully offset by increased employment in the renewable energy sector.

### Interpreting the divergent employment impacts

Our findings diverge significantly from a recent consensus in the literature, particularly studies directly examining China’s energy transition and employment, especially regarding whether the transition can reduce inequality. While some studies, such as the one by Zhang et al. [23], suggest that the energy transition has boosted employment in the tertiary sector and southeastern regions, implying a trend towards alleviating inequality, our results present a crucial counterpoint. In direct contrast to their conclusions, we find that China’s energy transition has triggered a “winner takes all” effect at both the sectoral and regional levels, which may actually exacerbate inequality. This finding is, however, consistent with a broader literature review by Godinho [17], which found that while the overall employment impact of climate policies is often moderate, neutral, or even positive, the distributional outcomes can be highly uneven, often to the detriment of certain groups and regions. Our empirical evidence on the “winner takes all” phenomenon provides a more granular and cautionary perspective on the distributional effects, empirically underscoring the significant transitional risks and costs to vulnerable regions and industries. We believe the robustness of our finding stems from the use of a multi-regional input-output model that, unlike single region or panel data studies, can explicitly and simultaneously capture the complex interregional labor migration and capital flows that drive this “winner takes all” effect, particularly in resource dependent regions.

Furthermore, our analysis of resource dependent regions adds a critical layer of nuance that is often overlooked in previous studies. We found that these regions, epitomized by Shanxi province, face an approximate 10% reduction in job opportunities due to the transition. This stark finding empirically reinforces the conceptual arguments made by scholars regarding the specific challenges faced by resource rich economies during a global shift away from fossil fuels.

### Contribution to knowledge and policy relevance

This study contributes to knowledge by bringing the social management consequences of decarbonization into carbon peak analysis. Existing carbon management studies have provided important evidence on low carbon pathways, provincial carbon budgets, economic development performance, coordinated emission reduction, and urban energy industry restructuring [1–6]. Building on this literature, our study adds a missing macroeconomic layer: it quantifies how a nationally desirable carbon peak pathway redistributes formal labor demand across sectors and regions. The main value added is therefore not a general description of labor market performance, but a management oriented warning that carbon peak pathways can create concentrated employment losses even when aggregate employment increases.

The results hold significant policy relevance for China and other developing nations. A carbon peak pathway should not be evaluated solely by aggregate emissions, GDP, or renewable energy expansion. It should also be assessed by its distributional feasibility: whether the regions that lose fossil fuel and heavy industrial employment have enough replacement opportunities, fiscal capacity, and retraining channels to absorb the shock. Our results show that renewable energy development is necessary but insufficient for a just transition because job creation in wind, photovoltaic, and other renewable sectors does not automatically occur in the same regions or among the same workers that bear fossil fuel employment losses. Therefore, carbon management should integrate regional compensation, industrial diversification, vocational retraining, and investment planning that accounts for supply chains into the design of carbon peak policies.

### Limitations and scope

To ensure our claims remain precise and defensible, it is crucial to clarify the scope and limitations of our modeling framework.

Scope: Our multi-regional input-output (MRIO) optimization model is specifically designed to capture the macro level structural reallocation of formal labor demand (urban unit employment) across 1,302 provincial and sectoral units under national carbon peaking constraints. It effectively traces how employment shocks in upstream resource dependent regions propagate through interregional supply chains.

Limitations: Readers should not overinterpret the results as absolute unemployment forecasts. The model does not account for demographic labor supply dynamics or informal sector employment. Furthermore, while we use provincial level data, tracking highly granular carbon footprints and exact indirect emission flows between specific cities remains outside our scope, an area that requires more specialized local carbon accounting models (Liu et al., 2026). Similarly, our scenario predictions rely on deterministic national parameters. Predicting exact peak timings at the localized urban level can be highly sensitive to specific municipal driving factors that our macro model does not capture [5].

Methodologically, the core assumption of wage rigidity, while justified by China’s employment stabilization policies, simplifies the dynamic wage adjustments of a perfectly competitive market. Additionally, our empirical baseline relies on the 2017 MRIO table. While it represents the most comprehensive cross regional data available, it may not fully capture the latest structural changes or the explicit policy interactions with China’s national Emissions Trading System. Future research should incorporate dynamic computable general equilibrium models and updated detailed datasets to address these limitations.

### Critical evaluation of theoretical assumptions

We acknowledge that our theoretical representation of the energy transition, primarily modeled as exogenous technological progress and accelerated fossil fuel phase out, is a high level macroeconomic abstraction. Real world energy transitions also encompass complex demand side behavioral shifts, power grid infrastructure upgrades, and intricate financial market dynamics. Our theoretical assumptions are deliberately parsimonious. They are designed strictly to isolate and evaluate the production side factor demand shocks (specifically, capital substituting labor and resources) rather than capturing the physical or engineering entirety of the transition. Consequently, these theoretical derivations should be interpreted as the fundamental economic mechanics driving labor reallocation, while the MRIO results provide the real world policy baseline.

## Conclusions and implications

In conclusion, this article has systematically explored how China’s national carbon peak transition reshapes the spatial and sectoral distribution of formal labor demand. Utilizing an integrated multi-regional input-output (MRIO) and multi-objective optimization model, the study shows that employment is a critical social management dimension of carbon peak pathways. The key message is that a transition pathway can be successful in aggregate carbon and economic terms while still producing severe regional and sectoral employment imbalances.

Our key findings are multifaceted: (1) The energy transition, while increasing total employment, creates a pronounced “winner takes all” effect at both regional and sectoral levels. Specifically, sectors and regions that already possess high economic output and technological advantages overwhelmingly monopolize the transition’s job creation benefits, leaving structurally vulnerable resource dependent areas further behind. Employment growth is concentrated in economically developed regions, while less developed and resource dependent areas face significant job losses. (2) Labor migration shows a clear trend from inland to coastal, and from northern to southern and eastern regions. (3) The decline in employment in traditional industries, particularly coal mining and power generation, is substantial and unlikely to be fully offset by the growth in renewable energy sectors. Although the number of jobs in renewable energy will more than quadruple, it is insufficient to directly fill the structural employment gap left by the coal sector. Unlike standard general equilibrium models that assume perfect labor mobility and constant total labor endowment, our empirical framework explicitly recognizes severe real world skill mismatches and geographic frictions. Displaced traditional miners cannot seamlessly transition into high tech renewable or tertiary sectors across different provinces, highlighting a profound structural vacancy rather than a simple algebraic reallocation.

Furthermore, the primary purpose of our theoretical policy analysis (Sect. 3) is to mathematically demonstrate that without deliberate structural intervention, capital biased and energy saving technological progress naturally leads to rent seeking and localized labor displacement. This theoretical foundation explicitly justifies why the Chinese government, which places a high priority on avoiding systemic unemployment, must rely on the extensive state led redistributive policies and vocational safety nets discussed below.

These findings hold substantial policy relevance for China and other developing economies navigating the complexities of the global energy transition. The clear existence of the “winner takes all” effect necessitates a proactive policy response to ensure a just transition. Key policy recommendations are structured around three critical dimensions: (1) Regions requiring targeted support: Our quantitative results indicate that resource dependent inland regions face severe and disproportionate labor displacement. For instance, Shanxi Province is projected to lose nearly 10% of its total urban unit employment opportunities (over 300,000 jobs). Policymakers must prioritize spatially targeted fiscal redistribution and transitional funds for these specific geographies to cushion the “winner takes all” shock [1]. (2) Sectors where risks concentrate: The structural transition poses acute risks to traditional fossil fuel sectors. Specifically, the coal mining and dressing industry faces extreme vulnerability, with a projected local workforce contraction of approximately 85%. Since these highly specialized workers cannot be seamlessly absorbed by the broader market, vocational training and early retirement programs must urgently and precisely target this demographic. (3) Relevant policy levers for job creation: While our simulations show that the renewable energy sector will expand its workforce by more than four times, this growth alone is insufficient to close the massive employment gap left by traditional sectors. As highlighted by recent transition pathway studies, policymakers must leverage broad structural shifts. The government should actively guide the displaced secondary sector workforce into high growth tertiary sectors such as transportation, warehousing, and leasing, which our model identifies as having the highest absolute job creation capacities per unit of added value.

## Data Availability

Data will be available upon request.

## Appendix A

See Tables 1, 2, 3, 4, 5, 6, 7, 8, 9 and 10.

**Table 1:** Industrial structure adjustment (annual adjustment rate, %)

Industrial structure adjustment intensity	Lower limit (%)	Upper limit (%)
Small	3.0%	7.0%
Big	5.0%	8.5%

**Table 2:** Scenario combination (annual growth rate, %)

Energy transformation intensity	Scenario no.	Year	Economic growth parameters	Energy transition parameters			Industrial structure parameters	
			GDP annual growth (%)	Energy consumption annual growth (%)	Carbon emission annual growth (%)	Coal growth (%)	Industrial shrinkage (%)	Industrial expansion (%)
Weak scenario of energy transformation	Scenario 1	2017–2025	7.50%	4.00%	4.50%	−3.00%	−5.00%	8.50%
		2026–2030	7.50%	4.00%	4.50%	−3.00%	−5.00%	8.50%
		2031–2037	7.50%	4.00%	4.50%	−3.00%	−5.00%	8.50%
	Scenario 2	2017–2025	6.50%	4.00%	4.50%	−3.00%	−3.00%	7.00%
		2026–2030	6.50%	4.00%	4.50%	−3.00%	−3.00%	7.00%
		2031–2037	6.50%	4.00%	4.50%	−3.00%	−3.00%	7.00%
Moderate scenario of energy transformation	Scenario 3	2017–2025	6.50%	3.30%	3.70%	−3.00%	−3.00%	7.00%
		2026–2030	6.50%	3.30%	3.70%	−3.00%	−3.00%	7.00%
		2031–2037	6.50%	3.30%	3.70%	−3.00%	−3.00%	7.00%
	Scenario 4	2017–2025	6.50%	3.30%	3.70%	−3.00%	−3.00%	7.00%
		2026–2030	7.00%	3.30%	3.70%	−3.00%	−5.00%	8.50%
		2031–2037	7.00%	3.30%	3.70%	−3.00%	−5.00%	8.50%
	Scenario 5	2017–2025	6.50%	3.30%	3.70%	−3.00%	−3.00%	7.00%
		2026–2030	7.00%	3.30%	3.70%	−3.00%	−5.00%	8.50%
		2031–2037	6.50%	3.30%	3.70%	−3.00%	−3.00%	7.00%
	Scenario 6	2017–2025	7.00%	3.30%	3.70%	−3.00%	−5.00%	8.50%
		2026–2030	7.00%	3.30%	3.70%	−3.00%	−5.00%	8.50%
		2031–2037	6.50%	3.30%	3.70%	−3.00%	−3.00%	7.00%
Strong scenario of energy transformation	Scenario 7	2017–2025	6.50%	3.00%	3.20%	−3.00%	−3.00%	7.00%
		2026–2030	6.50%	3.00%	3.20%	−3.00%	−3.00%	7.00%
		2031–2037	6.50%	3.00%	3.20%	−3.00%	−3.00%	7.00%
	Scenario 8	2017–2025	6.50%	3.00%	3.20%	−3.00%	−3.00%	7.00%
		2026–2030	7.00%	3.00%	3.20%	−3.00%	−5.00%	8.50%
		2031–2037	6.50%	3.00%	3.20%	−3.00%	−3.00%	7.00%

**Table 3:** Comprehensive summary of scenario constraints and core outputs (2017 to 2035)

Scenario no.	Theme/intensity	Key constraints (annual growth limits: GDP/energy/carbon)	Core outputs: peak year	Core outputs: 2035 emissions (billion tons)	Core outputs: 2035 GDP (trillion RMB)
S 1	Weak transition	High GDP (7.5%), High Energy (4.0%), High Carbon (4.5%)	2034	18.09	317.37
S 2	Weak transition	Med GDP (6.5%), High Energy (4.0%), High Carbon (4.5%)	No Peak	19.68	257.76
S 3	Moderate transition	Med GDP (6.5%), Med Energy (3.3%), Med Carbon (3.7%)	2035	17.13	252.13
S 4	Moderate transition	Dynamic GDP, Med Energy (3.3%), Med Carbon (3.7%)	2029	12.7	280.73
S 5	Moderate transition	Dynamic GDP, Med Energy (3.3%), Med Carbon (3.7%)	2029	13.45	266.4
S 6*	Optimal moderate	Dynamic GDP, Med Energy (3.3%), Med Carbon (3.7%)	2030	13.83	290.33
S 7	Strong transition	Med GDP (6.5%), Low Energy (3.0%), Low Carbon (3.2%)	2032	14.05	248.44
S 8	Strong transition	Dynamic GDP, Low Energy (3.0%), Low Carbon (3.2%)	2028	11.21	262.06

* Scenario 6 is identified as the optimal development path balancing economic cost and emission reduction

**Table 4:** Summary of carbon peak time for each scenario (GDP: trillion yuan; carbon emissions: 100 million tons CO2)

Scenario	Region	Peak or not	GDP (trillion yuan)	Peak year	Carbon emissions (100 million tons CO2)
Scenario1	Nation	Yes	317.37	2034	180.97
	Shanxi	Yes	4.82	2034	8.53
Scenario2	Nation	No	257.76	-	196.80
	Shanxi	No	4.26	-	7.81
Scenario3	Nation	Yes	252.13	2035	171.37
	Shanxi	No	4.16	-	7.74
Scenario4	Nation	Yes	182.01	2029	137.81
	Shanxi	Yes	3.20	2030	6.56
Scenario5	Nation	Yes	182.01	2029	137.81
	Shanxi	Yes	3.20	2030	6.56
Scenario6	Nation	Yes	215.18	2030	141.90
	Shanxi	Yes	3.57	2030	6.93
Scenario7	Nation	Yes	207.54	2032	142.93
	Shanxi	No	3.47	-	6.85
Scenario8	Nation	Yes	168.03	2028	122.15
	Shanxi	Yes	3.06	2029	6.30

**Table 5:** Estimation of employment changes in various sectors in China (unit: 10,000 persons)

Sector	2017 case (10,000 persons)	2035 case (10,000 persons)	Change in employment (10,000 persons)	Sector	2017 case (10,000 persons)	2035 case (10,000 persons)	Change in employment (10,000 persons)
1	255.40	216.26	−39.14	22	18.70	0.20	−18.50
2	304.30	28.04	−276.26	23	7.80	109.34	101.54
3	65.00	55.04	−9.96	24	12.40	0.20	−12.20
4	42.00	35.56	−6.44	25	377.00	1777.32	1400.32
5	44.00	37.26	−6.74	26	28.20	17.49	−10.71
6	224.30	189.92	−34.38	27	58.80	0.64	−58.16
7	170.80	88.38	−82.42	28	2643.20	1606.01	−1037.19
8	341.50	289.16	−52.34	29	842.80	1042.76	199.96
9	89.10	75.44	−13.66	30	843.90	2412.63	1568.73
10	231.80	120.17	−111.63	31	265.90	104.91	−160.99
11	62.10	5.72	−56.38	32	395.40	182.97	−212.43
12	244.40	22.52	−221.88	33	688.80	540.16	−148.64
13	350.40	32.29	−318.11	34	444.80	934.28	489.48
14	291.40	26.85	−264.55	35	522.60	1657.99	1135.39
15	164.10	138.95	−25.15	36	420.40	513.50	93.10
16	243.50	206.18	−37.32	37	268.50	227.35	−41.15
17	191.30	161.98	−29.32	38	75.40	63.84	−11.56
18	447.90	379.25	−68.65	39	1730.40	1465.19	−265.21
19	366.00	309.90	−56.10	40	897.90	760.28	−137.62
20	719.80	609.48	−110.32	41	152.20	128.87	−23.33
21	70.50	59.69	−10.81	42	1725.60	1461.12	−264.48

**Table 6:** Scale and change of employment in each province in 2035 (unit: 10,000 persons)

Province	Employment in 2017 (10,000 persons)	Employment in 2035 (10,000 persons)	Changes (10,000 persons)	Province	Employment in 2017 (10,000 persons)	Employment in 2035 (10,000 persons)	Changes (10,000 persons)
Beijing	671.50	749.09	77.59	Hubei	687.51	640.60	−46.91
Tianjin	390.16	432.65	42.49	Hunan	620.66	656.87	36.21
Hebei	722.24	737.11	14.87	Guangdong	1917.27	2126.47	209.20
Shanxi	389.15	358.67	−30.47	Guangxi	357.13	352.00	−5.12
Neimeng	423.31	404.67	−18.64	Hainan	86.45	83.63	−2.82
Liaoning	497.67	507.36	9.69	Chongqing	385.56	399.97	14.41
Jilin	289.53	306.93	17.40	Sichuan	724.54	668.88	−55.66
Heilongjiang	325.15	346.83	21.68	Guizhou	327.84	259.80	−68.04
Shanghai	585.98	648.69	62.71	Yunnan	382.85	319.66	−63.19
Jiangsu	1747.30	1878.68	131.38	Xizang	29.64	34.72	5.08
Zhejiang	1164.18	1227.01	62.83	Shannxi	403.71	419.98	16.28
Anhui	577.25	614.47	37.22	Gansu	180.92	153.56	−27.36
Fujian	710.63	782.39	71.76	Qinghai	68.07	52.00	−16.07
Jiangxi	393.09	378.25	−14.85	Ningxia	89.17	85.54	−3.63
Shandong	1373.62	1380.27	6.65	Xinjiang	263.54	271.61	8.07
Henan	854.41	816.75	−37.66

**Table 7:** Top 5 sectors and regions by employment change (ranked by absolute employment change)

Rank	Top 5 winning sectors	Top 5 losing sectors	Top 5 winning regions	Top 5 losing regions
1	Production and supply of electricity and heat	Buildings (Construction)	Guangdong	Guizhou
2	Transportation, warehousing and postal services	Non-metallic mineral products	Jiangsu	Sichuan
3	Leasing and business services	Coal mining and dressing products	Beijing	Yunnan
4	Real estate	Metal smelting and calendering products	Fujian	Hubei
5	Wholesale and retail	Chemical products	Zhejiang	Henan

**Table 8:** Renewable energy employment under the scenario of China’s carbon peak (unit: 10,000 persons, except generation proportion)

Year	Proportion of renewable energy power generation (%)	Wind power generation (10,000 persons)	Photovoltaic power generation (10,000 persons)	biomass power generation (10,000 persons)	Geothermal power generation (10,000 persons)	Hydrogen power generation (10,000 persons)	Total (10,000 persons)
2017	24%	53.66	31.01	9.02	20.41	17.41	131.52
2035	35%	137.82	79.65	23.18	52.43	44.72	337.80
	40%	157.50	91.03	26.49	59.92	51.11	386.06
	45%	177.19	102.41	29.80	67.41	57.50	434.32

**Table 9:** Employment scale of all departments in Shanxi Province in 2035 (unit: 10,000 persons)

No.	Employment in 2017 (10,000 persons)	Employment in 2035 (10,000 persons)	Changes (10,000 persons)	No.	Employment in 2017 (10,000 persons)	Employment in 2035 (10,000 persons)	Changes (10,000 persons)
1	2.07	2.54	0.47	22	0.02	0.00	−0.02
2	48.42	6.46	−41.96	23	0.78	0.95	0.18
3	0.25	0.30	0.06	24	0.00	0.00	0.00
4	0.61	0.75	0.14	25	105.83	79.48	−26.35
5	0.02	0.02	0.00	26	1.28	0.96	−0.32
6	1.24	1.52	0.28	27	0.01	0.01	0.00
7	0.09	0.07	−0.02	28	24.23	29.72	5.49
8	0.18	0.22	0.04	29	13.06	16.01	2.96
9	0.14	0.17	0.03	30	50.69	62.18	11.49
10	0.28	0.21	−0.07	31	1.86	2.28	0.42
11	1.60	0.21	−1.39	32	3.43	4.21	0.78
12	1.55	0.21	−1.34	33	9.52	11.68	2.16
13	2.36	0.32	−2.05	34	14.20	17.42	3.22
14	7.83	1.04	−6.78	35	13.30	16.32	3.01
15	1.05	1.29	0.24	36	3.24	3.98	0.73
16	0.71	0.87	0.16	37	2.80	3.44	0.63
17	0.95	1.16	0.21	38	1.17	1.44	0.27
18	1.17	1.43	0.26	39	26.45	32.44	5.99
19	1.00	1.23	0.23	40	9.00	11.03	2.04
20	4.58	5.61	1.04	41	2.08	2.56	0.47
21	0.16	0.20	0.04	42	29.96	36.75	6.79

**Table 10:** Employment scale of renewable energy in Shanxi Province in 2035 (unit: 10,000 persons, except generation proportion)

Year	Proportion of renewable energy power generation (%)	Wind power generation (10,000 persons)	Photovoltaic power generation (10,000 persons)	biomass power generation (10,000 persons)	Geothermal power generation (10,000 persons)	Hydrogen power generation (10,000 persons)	Total (10,000 persons)
2017	7.5%	0.53	0.31	0.09	0.20	0.17	1.31
2035	25%	4.40	2.54	0.74	1.67	1.43	10.79
	30%	5.28	3.05	0.89	2.01	1.71	12.95
	35%	6.16	3.56	1.04	2.34	2.00	15.11

## Appendix B

See Figs. 1, 2, 3 and 4.

## Appendix C: Mathematical derivations for the theoretical framework

Derivation of the critical capital-labor ratio (k̄) in short-term analysis: Given the labor marginal product FL = w = (1 − α)A(K/L)α. Taking the derivative with respect to α yields: dFL/dα = −A(K/L)α + (1 − α)A(K/L)α ln(K/L). Let dFL/dα = 0, and defining k = K/L, we get: −1 + (1 − α) ln(k) = 0 ⇒ ln(k) = 1/(1 − α) ⇒ k̄ = e(1/(1 − α)).

## Footnotes

1 It should be noted that, although we compare the energy transition to “labor saving technological progress”, this cannot change the technical coefficient of the energy. In fact, we assume that the energy efficiency is unchanged throughout the analysis, that is, the input-output ratio of energy is constant.

2 For Cobb Douglas production function $\:Y=A{K}^{\alpha\:}{L}^{1-\alpha\:}=A{\left(\frac{K}{L}\right)}^{\alpha\:}L$, take logarithms on both sides to get $\:lnL=lnY-\alpha\:ln\frac{K}{L}$.
